# Dr. Andrew Taylor Still

**Published:** 1918-02

**Authors:** 


**Dr. Andrew Taylor Still,** the founder of osteopathy, died Dec. 13, aged 89. Born in Philadelphia, he became a resident of Kansas before the Civil War, was elected to the legislature in 1859 on an anti-slavery platform and served in the war as a surgeon of the Union Army, reaching the rank of Major. He was a friend of John Brown. He broke away from regular medicine in 1874 and established the first school of osteopathy in 1892.

